# Radiation-induced FAP + fibroblasts are involved in keloid recurrence after radiotherapy

**DOI:** 10.3389/fcell.2022.957363

**Published:** 2022-08-24

**Authors:** Yan Gao, Xue Hou, Yuyin Dai, Ting Yang, Kexin Chen

**Affiliations:** ^1^ Department of Radiation Oncology and Therapy, The First Hospital of Jilin University, Changchun, China; ^2^ Department of Nuclear Medicine, The First Hospital of Jilin University, Changchun, China; ^3^ Laboratory of Cancer Precision Medicine, The First Hospital of Jilin University, Changchun, China; ^4^ Institute of Translational Medicine, The First Hospital of Jilin University, Changchun, China

**Keywords:** keloid scar, fibroblast activation protein alpha, radiation resistance, recurrence, xenotransplantation

## Abstract

**Background:** Keloid scars (KSs), which are composed of abnormal hyperplastic scar tissue, form during skin wound healing due to excessive fibroblast activation and collagen secretion. Although surgical resection and radiation therapy are used to prevent recurrence, KS recurrence rates range from 15 to 23%, and the underlying mechanism is unclear.

**Methods:** To elucidate the mechanism of keloid recurrence, we established a PDX model and the grafts remained for over 20 weeks after transplantation on the bilateral backs of the NCG mice.

**Results:** RNA-seq revealed that KS tissue gene expression was highly consistent before and after transplantation. Then, one side of the KS graft was irradiated with electron beam therapy (10 Gy), significant increases in vimentin and fibroblast activation protein alpha (FAP) expression were observed after irradiation and were accompanied by severe microvascular destruction. Surprisingly, 4 weeks after irradiation, significantly increased recurrence was observed with increased FAP + tissue and cell cycle regulator expression, resulting in a remarkable altered graft volume. Moreover, irradiation-induced FAP upregulation markedly facilitated radiation resistance and increased cell cycle progression, decreased senescence, and increased energy production.

**Conclusion:** Our findings revealed that irradiation causes increased abundance of FAP + cells, which was associated with cell proliferation and delayed cellular senescence, accompanied by ATP production.

## Introduction

Skin wound healing is an extremely complex process that includes the infiltration of inflammatory cells and secretion of growth factors and cytokines (Reinke and Sorg, 2012; Broughton, 2006). Keloid scars (KSs) are produced from a dermal fibroproliferative disorder that develops after burns, deep skin injuries and even surgical wounds (Ogawa, 2017). Although the mechanism of keloid formation is not fully understood, keloid is characterized by hyperthrophic fibroblast and collagen formation, angiogenesis and the upregulation of pro-inflammatory factors. Radiotherapy is currently recognized as one of the most important strategies for treating KSs (Mankowski et al., 2017). The favorable properties of electron irradiation make it the preferred choice for superficial keloid radiotherapy (Hogstrom and Almond, 2006; Hoppe, 2003; Maarouf et al., 2002). The recurrence rate with surgical resection alone, a traditional treatment for keloids and hypertrophic scars, ranges from 45% to 100% (Lee and Jang, 2018). Radiotherapy is often applied as a postoperative adjuvant therapy for keloids (Mustoe et al., 2002) and might control the rate of keloid recurrence to 15–23% (Mankowski et al., 2017). However, the mechanism remains unknown.

Since KSs occur only in humans, an appropriate animal model is necessary to explore new treatments and therapies. The major barrier to the engraftment of human-derived tissue in immune-competent rodents is robust xenogeneic immune rejection (Yang and Sykes, 2007). Fortunately, several strains of immunodeficient mice have been developed by disrupting relevant genes that are critical to the development, survival and function of immune cells (Marttala et al., 2016). In this study, we attempted to establish a novel PDX transplantation model using unique triple-immunodeficient mice designed using CRISPR-Cas9 technology to alter the *Prkdc* and *IL2rg* genes; the mice are more immunocompromised than commonly used immunodeficient mouse strains, such as SCID and nude mice. To study the mechanism of KS recurrence after surgical resection and electron beam treatment, human KS fragments without epidermal or dermal tissue were implanted into the backs of the NCG mice.

Fibroblast activation protein alpha (FAP) is a plasma membrane serine protease (dipeptidyl peptidase IV) that may play a key role in the invasiveness of keloids (Dienus et al., 2010). Normal adult tissues are generally negative for FAP, which exhibits both protease and collagenase activity and is important for extracellular matrix (ECM) degradation in wound healing and tumor invasion (O'Brien and O'Connor, 2008). These findings suggest that FAP may be a novel target for keloid treatment, but there have been few studies on the relationship between FAP and keloid recurrence after radiation therapy.

In this study, we evaluated the role of FAP in KS recurrence in an NCG xenograft mouse model. Although tissue loss occurred throughout the experiment, our data revealed that the graft was maintained in this new model for at least 20 weeks after implantation at the visible tissue level and that FAP + tissue was restored through radiation resistance, increased levels of cell cycle regulators, and high energy production.

## Materials and methods

### Ethical approval

Following approval of our protocol, which adhered to the ethical standards formulated in the Declaration of Helsinki, from the Institutional Ethics Committee of The First Hospital of Jilin University and the acquisition of written informed consent from all of the patients, KS tissues were obtained from 8 patients undergoing surgical excision.

### Tissue preparation and xenotransplantation

Briefly, immediately after surgical excision, the human KS mass was washed twice with phosphate-buffered saline and cut into 4-mm square pieces under sterile conditions. The mice were anaesthetized by intraperitoneal injection of 5% chloral hydrate with 0.1 ml/10 g body weight, and 1-cm incisions were made on both sides of the dorsal midline above the gluteus maximus. The human keloid tissues were implanted into the subcutaneous pocket between the panniculus carnosus and skin, and the wound was sutured.

Eight-week-old NCG mice were purchased from Charles River (Beijing, China) and bred in the specific pathogen–free (SPF) murine facility. The First Hospital of Jilin University Research Animal Care Committee (Changchun, Jilin) approved the entire process, and all the animals were kept under standard conditions described in the guidelines approved by the institution.

### Primary fibroblast isolation

KS tissues were washed twice with phosphate-buffered saline and cut into ∼1 mm3 sections under sterile conditions. The washed sections were placed in a culture plate with a distance of 1 cm between each section and incubated at 37°C for 30 min. The sections were then incubated in DMEM containing 10% FBS and antibiotics (penicillin, 100 U/ml; streptomycin, 0.1 mg/ml) at 37°C with 5% CO2. All cell culture reagents were supplied by Gibco (Thermo Fisher Scientific, Inc). The fibroblasts were trypsinized and prepared for subculture when they reached 90% confluency.

### Electron beam irradiation

At 4 weeks after implantation, irradiation therapy was performed with an electron beam (Clinac 21ex, Varian Medical System). The irradiation technique was consistent for all radiotherapies: The external beam was administered, with 6 MeV electrons generated by a linear accelerator. A single-fraction dose of 10 Gy (at a dose rate of 600 cGy/min) was delivered to the surgical incision with an electron beam. Nontarget areas were shielded using a 2-cm lead sheet.

### RNA preparation and KEGG pathway analysis

Degradation and contamination of the RNA from the samples were monitored on 1% agarose gels. RNA purity was checked by a Nano Photometer® spectrophotometer (IMPLEN, CA, United States). RNA integrity was assessed using the RNA Nano 6000 Assay Kit with the Bioanalyzer 2100 system (Agilent Technologies, CA, United States). The Kyoto Encyclopedia of Genes and Genomes (KEGG) database is a resource used to determine the high-level functions and roles of genes in biological systems. ClusterProfiler R packets were used to detect significant enrichment of differentially expressed genes in the KEGG pathway. Firstly, all significantly enriched terms and then calculated accumulative hypergeometric *p*-values and enrichment factors were identified and used for filtering. The remaining significantly enriched terms then underwent hierarchical clustering into a tree based on kappa-statistical similarities among the gene memberships. Then, a kappa score of 0.3 was applied as the threshold to divide the tree into term clusters.

### Quantitative real-time PCR

Total RNA was isolated with Total RNA Miniprep Kit (AP-MN-MS-RNA-250, Axygen), and cDNA was synthesized using 1st Strand cDNA Synthesis kit for qPCR (11123ES60, YEASEN Biotech, Shanghai). Quantitative RT-PCR was performed using Hieff® qPCR SYBR Green Master Mix (11203ES03, YEASEN Biotech, Shanghai) with a QuantStudio 5 RT qPCR system (ABI), and human-specific primer sets (Table 1) were obtained from Sangon Biotech (Shanghai, China). Beta-actin was used for normalization.

**TABLE 1:** qPCR primers specific for p21, p53, p16, Cyclin D1, CD34, FAP, vimentin and β-actin.

	Sense (5′-3′)	Antisense (5′-3′)
CD34	CCT​CAG​TGT​CTA​CTG​CTG​GTC​T	GGA​ATA​GCT​CTG​GTG​GCT​TGC​A
FAP	GGA​AGT​GCC​TGT​TCC​AGC​AAT​G	TGT​CTG​CCA​GTC​TTC​CCT​GAA​G
Vimentin	AGG​CAA​AGC​AGG​AGT​CCA​CTG​A	ATC​TGG​CGT​TCC​AGG​GAC​TCA​T
Cyclin D1	GCT​GCG​AAG​TGG​AAA​CCA​TC	CCT​CCT​TCT​GCA​CAC​ATT​TGA​A
p21	AGG​TGG​ACC​TGG​AGA​CTC​TCA​G	TCC​TCT​TGG​AGA​AGA​TCA​GCC​G
P53	CCT​CAG​CAT​CTT​ATC​CGA​GTG​G	TGG​ATG​GTG​GTA​CAG​TCA​GAG​C
p16	CTC​GTG​CTG​ATG​CTA​CTG​AGG​A	GGT​CGG​CGC​AGT​TGG​GCT​CC
β-Actin	ATT​GCC​GAC​AGG​ATG​CAG​AAG	CCA​TGC​CAA​TCT​CAT​CTT​GT

### Histology and IHC

Tissues were collected, fixed with 4% formalin and embedded in paraffin. The paraffin sections (4 μm) were stained with H&E or subjected to IHC. Briefly, the tissue sections were subjected to antigen retrieval and incubation with primary antibodies against FAP (AF5344, Affinity), vimentin (AF7013, Affinity), or CD34 (AF5149, Affinity), followed by incubation with secondary antibody (KIT-9706, Maixin-Bio), and immunoreactivity was detected using an Peroxidase Kit (KIT-9710, Mai Xin). Quantitative analysis was performed using Image-Pro Plus 6.0 software.

### Cell proliferation assay

Cell proliferation was assessed using a Cell Counting Kit-8 (CCK-8) assay kit (C0038, Beyotime) according to the manufacturer’s protocol. Briefly, KFs (3×10^3^ cells per well) were seeded into a 96-well plate and cultured at 37°C in a 5% CO2 incubator. CCK-8 solution was added for 2 h and the absorbance was measured at 490 nm.

### SA-β-gal activity analysis

SA-β-gal active assay was performed as our previous study using an SA-β-gal staining kit (Beyotime, Beijing). Briefly, KFs were plated in 12-well plates (6×10^4^/well) and were irradiated with 10 Gy and analyzed upon reaching 80–90% confluence. SA-β-gal-positive cells were identified as green-stained cells and the frequency was determined by counting approximately 400 cells in three random fields.

### Flow cytometry

To measure radiation-induced cytomembrane expression of FAP or cytoplasmic expression of vimentin, primary KFs were irradiated with a single-fraction dose of 10 Gy (at a dose rate of 600 cGy/min) with an electron beam. The cells were analyzed by staining with an AF488-conjugated anti hFAP antibody (R&D Systems, FAB3715G-100) and a PE-conjugated anti-hVimentin antibody (BD, 562337). FAP+ KFs were sorted according to staining with the AF488-conjugated antihFAP antibody (BD FACSCalibur) at 72 h after irradiation.

### Measurement of intracellular ATP levels

Intracellular basal ATP production rates were measured using Seahorse XF Real-Time ATP Rate Assay Kit (Agilent). This assay was performed according to the manufacturer’s protocol. Briefly, KF cells were seeded into 24-well plates with 3 × 104 cells per well and incubated at 37°C with 5% CO2 for several days. One hour after medium was replaced by indicated medium without bicarbonate, the OCR and ECAR were measured with XF24 (Seahorse Bioscience, United States) before and after injection of 1.5 μM oligomycin, 0.5 μM rotenone and 0.5 μM antimycin. The basal ATP production rates were compared by mitoATP Production Rate and glycoATP Production Rate.

### Statistical analysis

All studies were repeated at least three times and data are presented as the mean ± SD. Statistical analysis was performed using Prism 6 (GraphPad Software). One-way ANOVA was used for comparison of multiple groups, while the *t* test was used for comparison of two groups. *p* < 0.05 were taken as significant.

## Results

### Establishment of a patient-derived KS tissue xenotransplantation model using NCG mice and morphological observation

**FIGURE 1:**
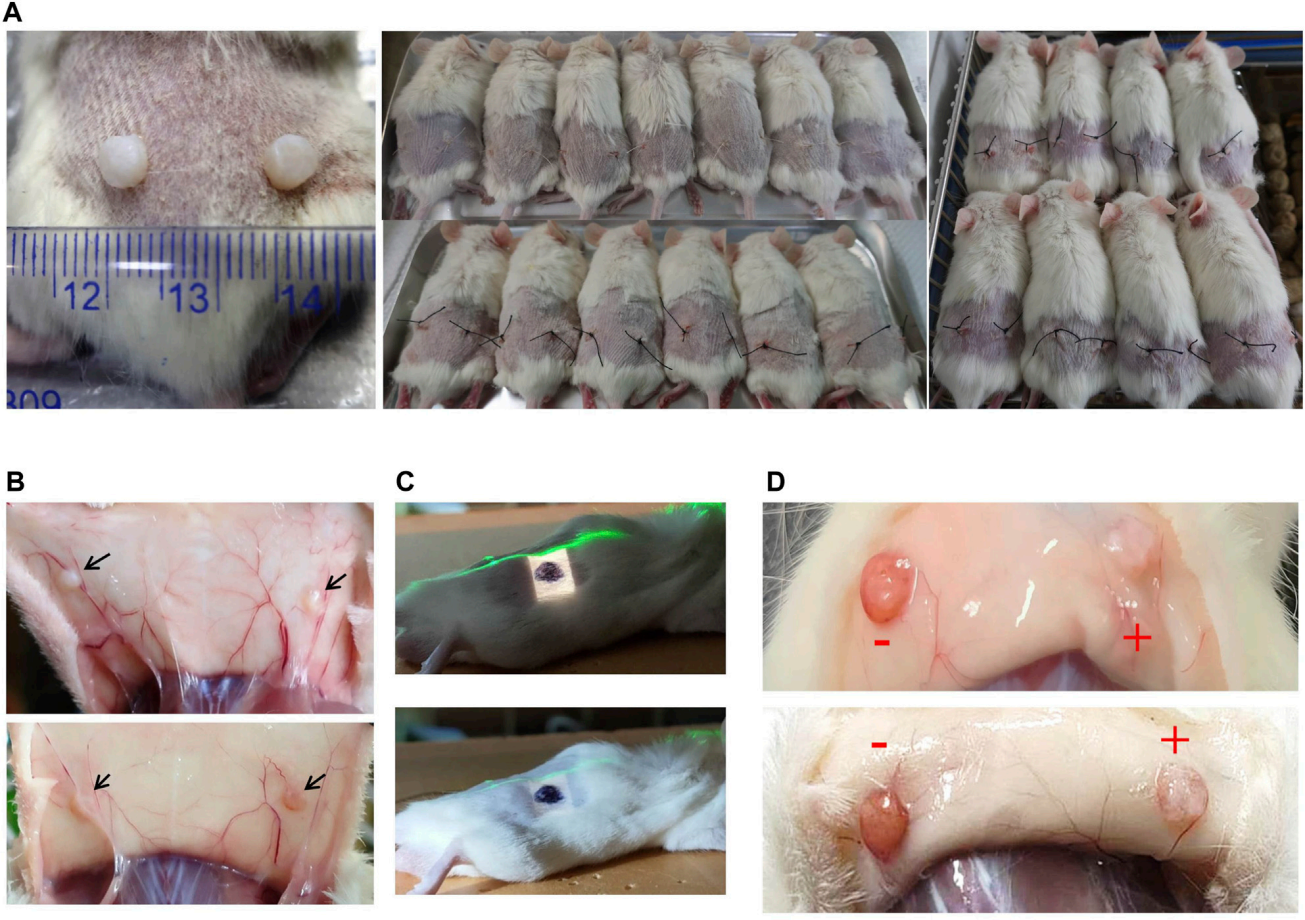
Xenotransplantation of patient-derived KS tissue in NCG mice and radiation therapy. **(A)** The grafts and implantation positions of the grafts are shown. **(B)** The grafts were visible at 20 weeks after implantation without irradiation. Representative images are shown. **(C)** Image of the field irradiated with an electron beam. The right side of the graft is marked in black. The green line is the laser alignment line. **(D)** One week after irradiation, graft morphological alterations were observed. Representative images are shown.

To establish a PDX mouse model to study KSs, human KS tissues (from 8 patients) were cut into 3–4-mm square pieces and implanted into the backs of NCG mice (>60) under sterile conditions (Figure 1A). Previous studies have shown that KS grafts can be maintained in nude mice for at least 4 months after operation, as shown by detection at the cellular level (Park et al., 2016; Shetlar et al., 1985). In this study, the grafts were still visible at 20 weeks after transplantation, although significant tissue loss was observed (Figure 1B). According to our previous work, more than 3 weeks are needed for the graft to establish stable neovascularization. At 4 weeks after implantation, the right sides of the grafts in the KS-bearing mice were irradiated with a single-fraction dose of 10 Gy with an electron beam (Figure 1C). One week after irradiation, morphological alterations in the graft were observed. As shown in Figure 1D, in contrast to nonirradiated grafts (−), the irradiated grafts showed evidence that the electron beam had severely destroyed the neovascularization (+). Taken together, these findings demonstrated that we successfully established a PDX mouse model of KSs using NCG mice and observed severe electron beam-induced damage to the vascular network of the graft.

### Irradiation induced microvascular destruction and tissue loss

**FIGURE 2:**
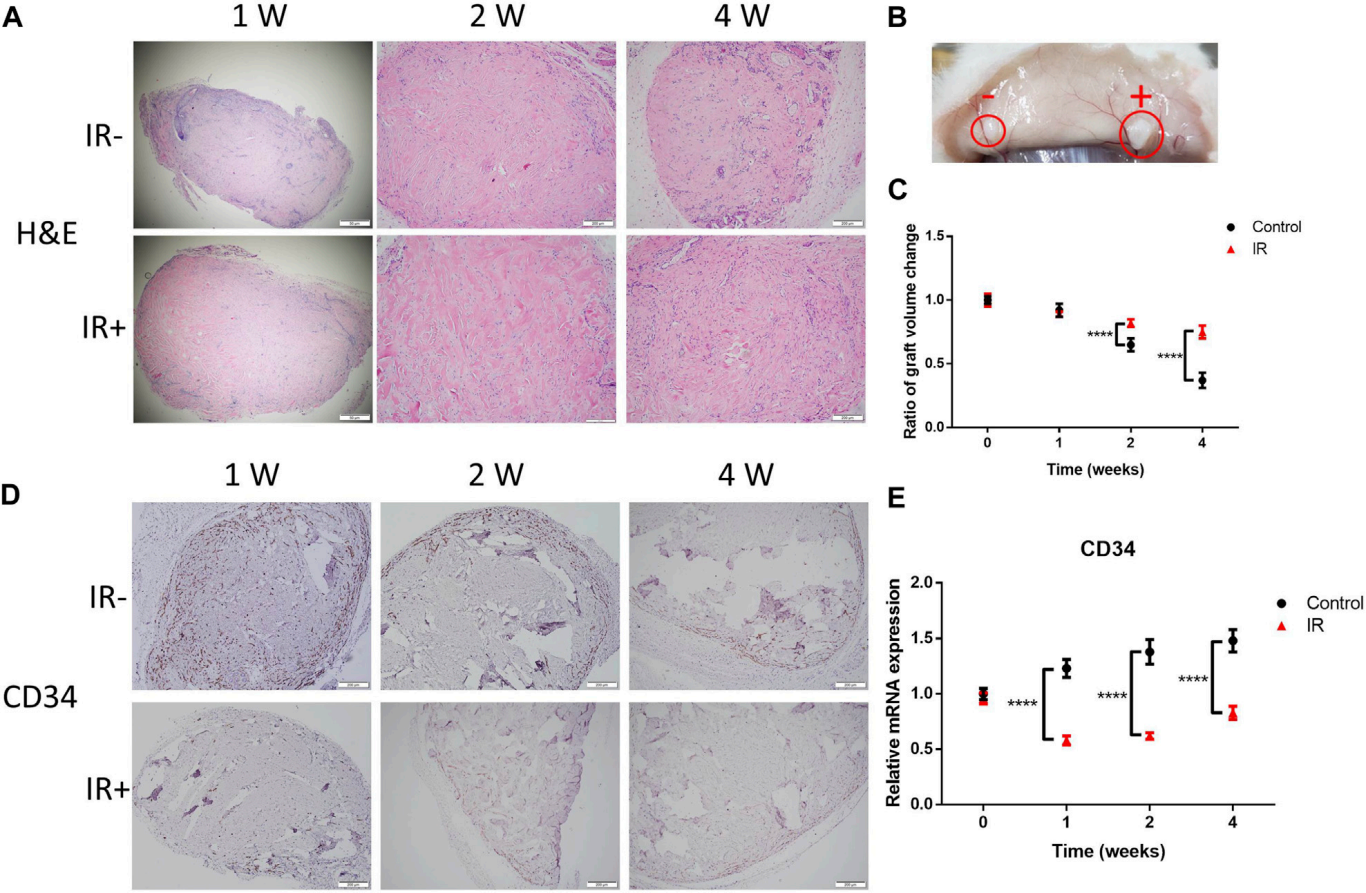
Irradiation (IR) induced destruction of the microvasculature and tissue loss. **(A)** Representative images showing H&E staining, **(B)** a morphological comparison at 4 weeks after IR and **(C)** a comparison of the graft volume ratio. Images from 6 representative random fields are shown. The scale bars represent 200 µm except for the scale bar of 1 W in **(A)**, which represents 50 µm. **(D)** Graft sections were stained for CD34 for IHC analysis. Six samples per group were examined, and representative images are shown (scale bar represents 200 μm). **(E)** Comparison of the CD34 levels in the two groups of grafts. Data from 3 experiments were combined and are presented as the mean ± SD; *n* = 6; ****, *p* < 0.001.

To investigate the effect of the electron beam on implanted KS tissue and determine whether irradiation can suppress the development of graft tissue, histological analysis was performed. As shown in Figure 2A, H&E staining of the grafts was performed at 1, 2 and 4 weeks after irradiation. The electron beam induced sharp decreases in the numbers of infiltrating cells in the graft tissues, especially during the first week after irradiation (Figure 2A, left row). Surprisingly, irradiation disrupted the vascular network of the graft but slowed the process of tissue loss (Figures 2A,B). At 4 weeks after irradiation, the volume ratio of the irradiated grafts was significantly larger than that of the control grafts by over twofold (0.75 vs. 0.37). Moreover, histological analysis was performed to determine whether the electron beam would destroy the neovascularization in the graft tissue (Figures 2D,E). Consistent with the results in Figure 1C, our results revealed that irradiation induced a sharp decrease in microvascular density and CD34 mRNA levels.

### Irradiation destroyed the internal structure of KS tissues, but FAP+ and vimentin + tissues were quickly restored

**FIGURE 3:**
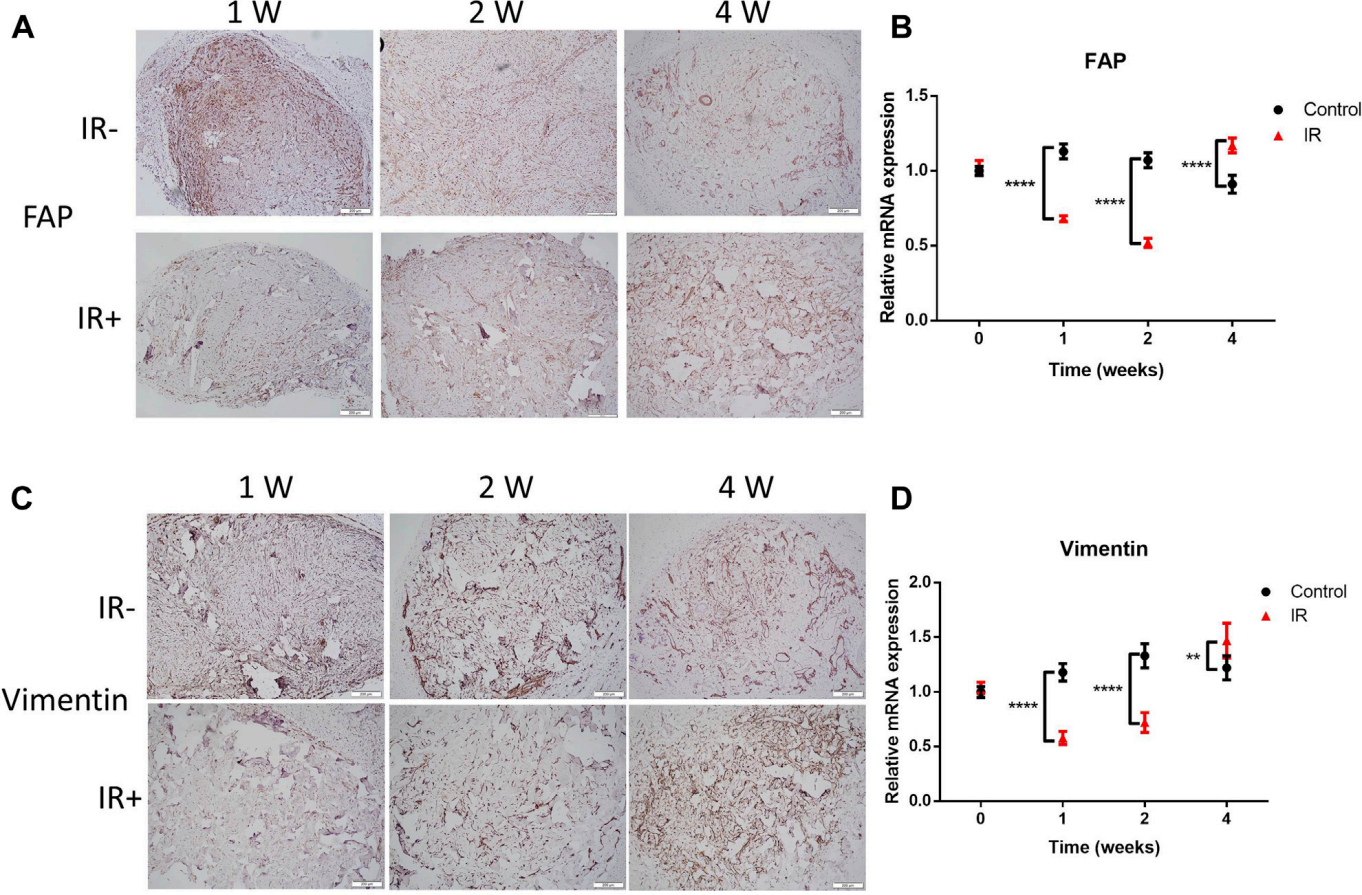
Radiation-resistant tissue was quickly restored, with increased FAP and vimentin expression. **(A–C)** Graft sections were stained for FAP and vimentin for IHC analysis. The scale bar represents 200 µm. **(B–D)** Comparison of the mRNA levels of FAP and vimentin between the two groups of grafts. Data from 3 experiments were combined and are presented as the mean ± SD. *n* = 6; **, *p* < 0.01; ****, *p* < 0.001.

Fibroblasts are thought to play a key role in fibrogenesis in KSs. FAP is a plasma membrane-localized protease associated with the development of KSs (Dienus et al., 2010). To determine whether irradiation could promote the expression of FAP in graft tissue, histological analysis was performed. As shown in Figure 3A, the electron beam significantly increased the FAP level compared to that in the control graft after irradiation. This result was consistent with the mRNA level of FAP (Figure 3B). Increased levels of vimentin were reported to serve as a mesenchymal marker, and the frequency of vimentin **+** epidermal cells was found to be higher in KSs and keloid microvessels than in normal skin (Yan et al., 2015; Hahn et al., 2016). Therefore, we observed changes in vimentin expression in the grafts after irradiation. Surprisingly, the change in vimentin expression beginning in the second week after irradiation was dramatically reversed (Figures 3C,D). These results suggest that irradiation damaged FAP+ and vimentin + tissue in the grafts but that the levels of both molecules were significantly restored over 3 weeks. This finding is consistent with the observed changes in graft volume after irradiation (Figure 2B).

### Irradiation significantly upregulated cell cycle regulators

**FIGURE 4:**
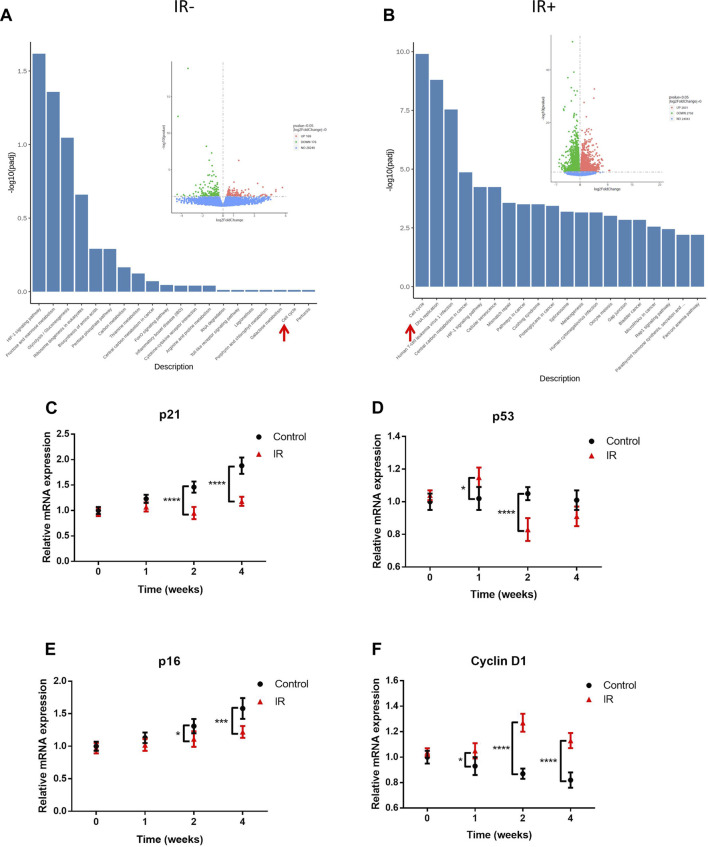
Irradiation (IR) promoted cell cycle regulator expression in radiation-resistant tissue. RNA-seq analysis of nonimplanted KS tissue compared with unirradiated tissue at 4 weeks after implantation **(A)** and irradiated tissue at 4 weeks after irradiation **(B)**. The insert from the volcano map indicates the number of gene alterations. The columns are KEGG pathways, and the spots are the number of genes. Green indicates downregulation, and red indicates upregulation. The red arrow indicates the cell cycle pathway. Shown are the mRNA levels (mean ± SD; n = 6) of the CKIs p21/p53/p16 **(C–E)** and the promoter Cyclin D1 **(F)**. Data from three experiments with essentially the same results were combined and are presented as the mean ± SD; *n* = 6; *, *p* < 0.05; ***, *p* < 0.005; ****, *p* < 0.001.

To clarify the effects of transplantation and irradiation on KSs, we compared the transcriptome activity of nonimplanted KS tissue with that of KS tissue at 4 weeks after transplantation (Figure 4A) and 4 weeks after irradiation (Figure 4B). Compared to the control, in our PDX model, only 169 genes were upregulated, and 176 genes were downregulated, despite the 4-week experimental period (Figure 4A insert). However, irradiation induced a large change in expression, with 2831 genes upregulated and 2758 genes downregulated compared to their expression in the PDX model (Figure 4B, insert). Moreover, KEGG analysis revealed that the expression of cell cycle regulators was significantly increased by irradiation. Furthermore, the mRNA expressions were compared for the key cell cycle inhibitors (cyclin-dependent kinase (CDK) inhibitors, CKIs) p21, p53 and p16, which are known tumor suppressor proteins that are upregulated in irradiated tissue (Aratani et al., 2018). Consistent with the immunohistochemistry (IHC) results (Figure 3), expression of the CKIs p53, p21 and p16 after 4 weeks was significantly reduced by irradiation compared with that in the control (Figures 4C–E). Interestingly, the expression of p53, the primary guardian of the DNA damage response, was significantly upregulated 1 week after irradiation compared with that in the control group but sharply downregulated 1 week later (Figure 3B). On the other hand, irradiation significantly increased the level of Cyclin D1 (Figure 4F), a cell cycle promoter that regulates cell cycle progression by phosphorylating CDK4/6 to inhibit retinoblastoma (Connell-Crowley et al., 1997). Taken together, these results suggested that radiation-resistant tissue (some of which may be positive for FAP) among KS tissue may cause KS recurrence due to irradiation.

### FAP + keloid fibroblasts (KFs) promoted cell cycle regulator expression in radiation-resistant tissue with increased energy production

**FIGURE 5:**
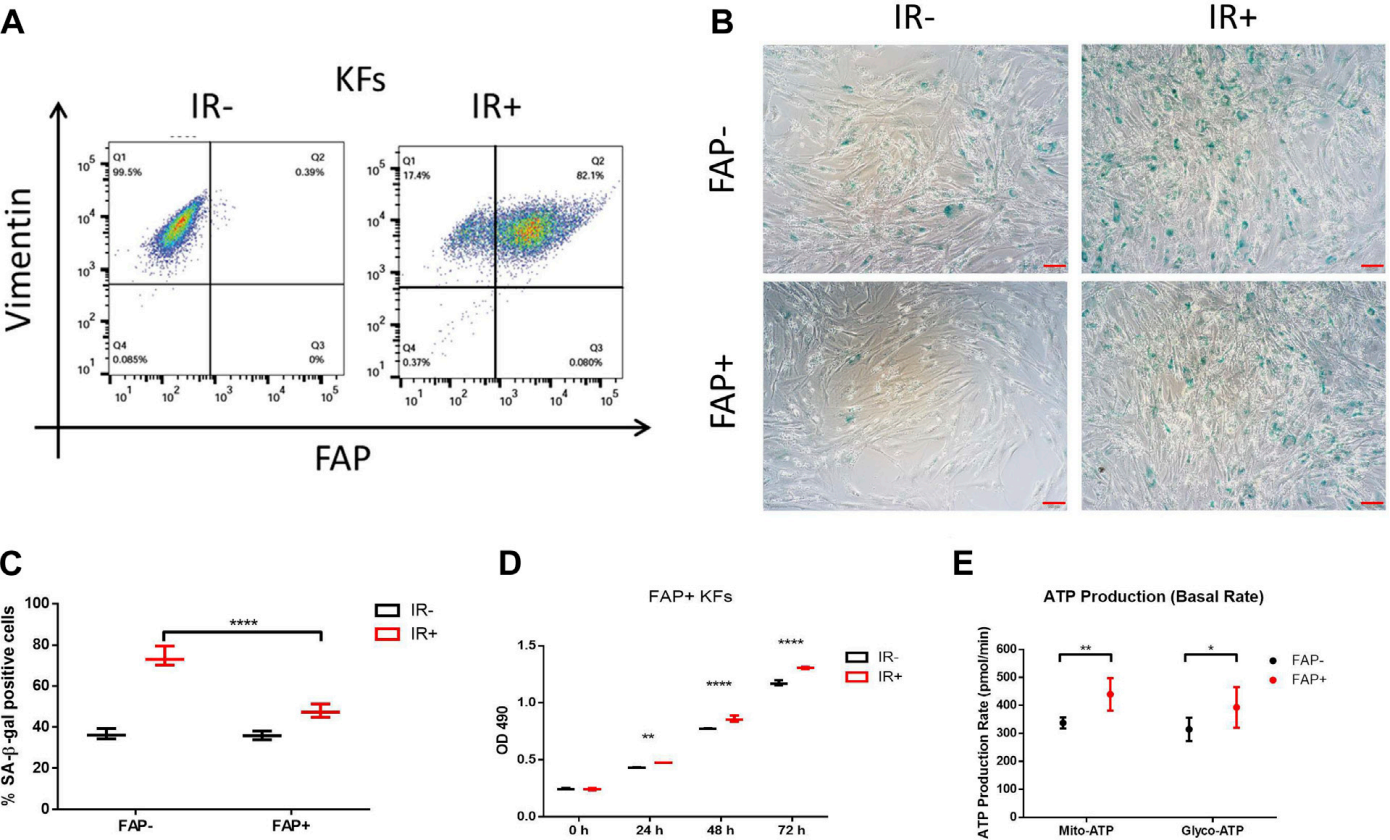
Irradiation (IR) promoted FAP + KF proliferation and inhibited senescence, accompanied by high energy production. *In vitro*, primary KFs with or without IR treatment were analyzed by anti-hFAP and anti-hVimentin antibody staining **(A)**. Representative images of SA-β-gal staining **(B)**; senescent cells are stained green, scale bar = 100 μm. The percentage of senescent cells in FAP- versus FAP + KFs at 72 h after IR **(C)**. Data are representative of three independent samples and are presented as the mean ± SD; *n* = 6; ****, *p* < 0.001. IR promoted FAP + KF proliferation **(D)** and increased intracellular ATP production **(E)**. Data from three experiments with essentially the same results were combined and are presented as the mean ± SD; *n* = 3; *, *p* < 0.05; **, *p* < 0.01; ****, *p* < 0.001.

Our data indicated that FAP + tissue could be quickly restored *in vivo*. To investigate whether irradiation could induce FAP + cell proliferation, primary KFs were isolated from patient excisions and irradiated with 10 Gy electron beam therapy. Vimentin, a fibroblast biomarker, is also known as fibroblast intermediate filament in non-muscle cells. The flow cytometry data revealed that the isolated primary KFs were 99.5% vimentin positive. Surprisingly, radiation induced a significant increase in the FAP + KF population; the FAP + KF percentage in the irradiation group was 82.1%, while that in the non-irradiation group was 0.39% (Figure 5A). To determine the role of FAP in radiation-induced senescence, the FAP + KFs were sorted, and senescence-associated β-galactosidase (SA-β-gal) activity was measured at 72 h after irradiation. Irradiation clearly increased the percentages of SA-β-gal + cells among both types of KFs; however, the increase was markedly smaller in the FAP + group than in the FAP- group, and the percentage of SA-β-gal + cells was significantly lower in the FAP + group than in the FAP- group (Figures 5B,C). Although the numbers of viable cells were comparable between nonirradiated and irradiated KF cultures at 0 h, the irradiated cultures thereafter yielded significantly more viable cells (Figure 5D). The observation of rapid recurrence with increased FAP expression and cell proliferation after irradiation suggests the need for an adequate energy supply. Adenosine triphosphate (ATP), the most common cellular energy currency of intermediate metabolism, is used to sustain various cellular functions such as anabolic synthesis, molecular transport, cell motility, and cell proliferation. Thus, ATP levels were measured to compare FAP+ and FAP- KF cell viability (Figure 5E). FAP + KFs had significantly higher ATP concentrations and showed a significantly greater rate of ATP production from glycolysis (393.1 vs. 314.8 pmol/min) and mitochondrial oxidative phosphorylation than FAP- KFs (439.8 vs. 337.5 pmol/min). Taken together, our results demonstrate a strong association between FAP + KF abundance and radiation resistance through cell cycle progression and increased energy production.

## Discussion

Keloid, a fibroproliferative disease and physiological phenomenon unique to humans, can occur in genetically susceptible individuals (Brown and Bayat, 2009). Thus, because keloid is unique to humans, it is difficult to conduct *in vivo* studies through the use of animal models. Fortunately, the development of immunodeficient mice strains might provide a novel approach to investigate the pathological progression of KSs *in vivo* by establishing reliable patient-derived KS xenografts models that remain viable for several weeks to months after transplantation (Marttala et al., 2016; Shultz et al., 2007). In this study, we established a PDX model of human-derived KS tissue by using NCG mice, and the graft successfully survived *in vivo* for as long as 20 weeks. Although significant tissue loss was observed over time, all grafts retained their original histotypic and morphological characteristics for 20 weeks, providing a sufficiently long window to investigate preclinical therapies and molecular mechanisms.

Numerous studies have shown the favorable effects of electron beam radiotherapy for keloid treatment. For example, recent data revealed that surgical excision followed by electron beam radiation provides excellent local control of keloids and reduces the recurrence rate to 8.6% (Wang et al., 2020), while the recurrent rate for traditional keloid surgery alone is up to 45–100% (Lee and Jang, 2018). Regarding optimal irradiation timing, most studies recommended electron beam radiotherapy as an adjuvant therapy within 24–48 h after keloid revision surgery (Ogawa et al., 2003). Mechanistically, the fibroblasts that dominated the incisional granulation tissue at this time were found to be sensitive to irradiation. Therefore, this radiotherapy strategy can effectively suppress the cell proliferation and division of fibroblasts and reduce collagen deposition. However, no consensus for the optimal irradiation dose and fraction regimen has been reached. In previous studies, keloid treatment has tended to involve postoperative irradiation with a dose of 12–20 Gy over 3–5 fractions (Renz et al., 2018)^,^ (Bischof et al., 2007). Herein, radiation with 20 Gy over 5 fractions was confirmed to yield superior local control compared to the control achieved with lower-dose regimens (Renz et al., 2018). Recently, emerging studies have demonstrated that the use of hypofractionated postoperative radiotherapy with a high-energy electron beam for keloids is an excellent strategy and that initiating radiotherapy as soon as possible after surgery could improve the therapeutic regimen (Shen et al., 2015). Adjuvant single-fraction radiotherapy with a dose of 8–10 Gy from an electron beam was also confirmed to be effective and safe for treating keloids (Song et al., 2014), (Sruthi et al., 2018). Concerning unresectable keloids, previous data have demonstrated that radical radiotherapy can achieve satisfactory and similar efficacy (Malaker et al., 2004). As a result, postsurgical radiotherapy has become the most widely accepted method for the treatment of keloids.

Here, we attempted to irradiate our PDX model of human-derived KS tissue with an electron beam. The results revealed that irradiation quickly destroyed the neovascular system of the graft and significantly reduced levels of the KS-related factors FAP and vimentin (Figures 2, 3). Interestingly, similar changes in graft volume and in FAP and vimentin were observed, and KS relapse was accompanied by restored FAP and vimentin expression. FAP, a proline-selective serine protease, is overexpressed in hypertrophic/KS tissue and the tumor stroma (cancer/tumor-associated fibroblasts, CAFs/TAFs) but undetectable in most normal adult tissues (Kalluri and Zeisberg, 2006; Spaeth et al., 2009). FAP plays an important role in ECM remodeling via its serine protease activity (Kennedy et al., 2009). Tumor relapse is usually caused by radiation resistance, which is determined by both the intrinsic characteristics and external microenvironment of cancer cells (Wang et al., 2017; Wang et al., 2005). Accumulating evidence has revealed that FAP + CAFs might provide a more immunosuppressive microenvironment to resist damage from immunocytes/radiotherapy/chemotherapy, resulting in increased tumor survival (Yang et al., 2016; Lindner et al., 2018; Ansems and Span, 2020). Moreover, a similar result from a transplanted tumor model demonstrated that FAP + stromal cells could facilitate immunosuppression via ablation of T cell antitumor activity (Kraman et al., 2010). However, the mechanism of induction of FAP expression remains unclear. Here, our data revealed that irradiation by electron beam could promote the FAP level *in vitro* and *in vitro* for the first time, and the reduction of FAP + tissue in the control group was positively correlated with tissue loss (Figure 3A), while the rebound of FAP + tissue in the irradiated group was also proportional to graft volume. In addition, our transcriptome experiments revealed that irradiation significantly enhanced translational activity in the irradiated grafts, including the translation of cell cycle regulators. Cyclin D1 plays a key role in regulating the G1/S transition by activating CDKs, whereas CKIs, such as p21 and p16, inhibit the cyclin/CDK complex kinase activity. Our data revealed that irradiation promoted cell cycle progression in FAP + cells, accompanied by upregulation of Cyclin D1 and downregulation of the CKIs p53, p21 and p16. In this study, we discovered that the induction of FAP expression by electron beam only in the primary human keloid derived fibroblast, we also attempted to detect radiation-induced FAP expression in mouse fibroblast cell line, but the cell line displayed a poor radio-sensitivity (data not shown). The role of enzymatic activity and ablation of FAP in recurrence of KS will be further investigated.

Taken together, our findings revealed a prominent expression of FAP in KS recurrence after surgical resection and radiation therapy, indicating that irradiation might enhance FAP + cell abundance, which was associated with cell proliferation and delayed cellular senescence, accompanied by ATP production.

## Data Availability

The raw data supporting the conclusions of this article will be made available by the authors, without undue reservation.

## References

[B1] Ansems M. Span P. N. (2020). The tumor microenvironment and radiotherapy response; a central role for cancer-associated fibroblasts. Clin. Transl. Radiat. Oncol. 22, 90–97. 10.1016/j.ctro.2020.04.001 32337377 PMC7177030

[B2] Aratani S. Tagawa M. Nagasaka S. Sakai Y. Shimizu A. Tsuruoka S. (2018). Radiation-induced premature cellular senescence involved in glomerular diseases in rats. Sci. Rep. 8 (1), 16812. 10.1038/s41598-018-34893-8 30429495 PMC6235850

[B3] Bischof M. Krempien R. Debus J. Treiber M. (2007). Postoperative electron beam radiotherapy for keloids: Objective findings and patient satisfaction in self-assessment. Int. J. Dermatol. 46 (9), 971–975. 10.1111/j.1365-4632.2007.03326.x 17822505

[B4] Broughton G. Janis J. E. Attinger C. E. (2006). Wound healing: An overview. Plast. Reconstr. Surg. 117 (7), 1e-S–32e-S. 10.1097/01.prs.0000222562.60260.f9 16801750

[B5] Brown J. J. Bayat A. (2009). Genetic susceptibility to raised dermal scarring. Br. J. Dermatol. 161 (1), 8–18. 10.1111/j.1365-2133.2009.09258.x 19508304

[B6] Connell-Crowley L. Harper J. W. Goodrich D. W. (1997). Cyclin D1/Cdk4 regulates retinoblastoma protein-mediated cell cycle arrest by site-specific phosphorylation. Mol. Biol. Cell 8 (2), 287–301. 10.1091/mbc.8.2.287 9190208 PMC276080

[B7] Dienus K. Bayat A. Gilmore B. F. Seifert O. (2010). Increased expression of fibroblast activation protein-alpha in keloid fibroblasts: Implications for development of a novel treatment option. Arch. Dermatol. Res. 302 (10), 725–731. 10.1007/s00403-010-1084-x 20872224

[B8] Hahn J. M. McFarland K. L. Combs K. A. Supp D. M. (2016). Partial epithelial-mesenchymal transition in keloid scars: Regulation of keloid keratinocyte gene expression by transforming growth factor-β1. Burns Trauma 4 (1), 30. 10.1186/s41038-016-0055-7 27574697 PMC4994224

[B9] Hogstrom K. R. Almond P. R. (2006). Review of electron beam therapy physics. Phys. Med. Biol. 51 (13), R455–R489. 10.1088/0031-9155/51/13/R25 16790918

[B10] Hoppe R. T. (2003). Mycosis fungoides: Radiation therapy. Dermatol. Ther. 16 (4), 347–354. 10.1111/j.1396-0296.2003.01647.x 14686978

[B11] Kalluri R. Zeisberg M. (2006). Fibroblasts in cancer. Nat. Rev. Cancer 6 (5), 392–401. 10.1038/nrc1877 16572188

[B12] Kennedy A. Dong H. Chen D. Chen W. T. (2009). Elevation of seprase expression and promotion of an invasive phenotype by collagenous matrices in ovarian tumor cells. Int. J. Cancer 124 (1), 27–35. 10.1002/ijc.23871 18823010 PMC2597700

[B13] Kraman M. Bambrough P. J. Arnold J. N. Roberts E. W. Magiera L. Jones J. O. (2010). Suppression of antitumor immunity by stromal cells expressing fibroblast activation protein-alpha. Science 330 (6005), 827–830. 10.1126/science.1195300 21051638

[B14] Lee H. J. Jang Y. J. (2018). Recent understandings of biology, prophylaxis and treatment strategies for hypertrophic scars and keloids. Int. J. Mol. Sci. 19 (3), 711. 10.3390/ijms19030711 PMC5877572 29498630

[B15] Lindner T. Loktev A. Altmann A. Giesel F. Kratochwil C. Debus J. (2018). Development of quinoline-based theranostic ligands for the targeting of fibroblast activation protein. J. Nucl. Med. 59 (9), 1415–1422. 10.2967/jnumed.118.210443 29626119

[B16] Maarouf M. Schleicher U. Schmachtenberg A. Ammon J. (2002). Radiotherapy in the management of keloids. Clinical experience with electron beam irradiation and comparison with X-ray therapy. Strahlenther. Onkol. 178 (6), 330–335. 10.1007/s00066-002-0935-6 12122789

[B17] Malaker K. Vijayraghavan K. Hodson I. Al Yafi T. (2004). Retrospective analysis of treatment of unresectable keloids with primary radiation over 25 years. Clin. Oncol. 16 (4), 290–298. 10.1016/j.clon.2004.03.005 15214654

[B18] Mankowski P. Kanevsky J. Tomlinson J. Dyachenko A. Luc M. (2017). Optimizing radiotherapy for keloids: A meta-analysis systematic review comparing recurrence rates between different radiation modalities. Ann. Plast. Surg. 78 (4), 403–411. 10.1097/SAP.0000000000000989 28177974

[B19] Marttala J. Andrews J. P. Rosenbloom J. Uitto J. (2016). Keloids: Animal models and pathologic equivalents to study tissue fibrosis. Matrix Biol. 51, 47–54. 10.1016/j.matbio.2016.01.014 26827712 PMC4842112

[B20] Mustoe T. A. Cooter R. D. Gold M. H. Hobbs F. D. Ramelet A. A. Shakespeare P. G. (2002). International clinical recommendations on scar management. Plast. Reconstr. Surg. 110 (2), 560–571. 10.1097/00006534-200208000-00031 12142678

[B21] O'Brien P. O'Connor B. F. (2008). Seprase: An overview of an important matrix serine protease. Biochim. Biophys. Acta 1784 (9), 1130–1145. 10.1016/j.bbapap.2008.01.006 18262497

[B22] Ogawa R. (2017). Keloid and hypertrophic scars are the result of chronic inflammation in the reticular dermis. Int. J. Mol. Sci. 18 (3), 606. 10.3390/ijms18030606 PMC5372622 28287424

[B23] Ogawa R. Mitsuhashi K. Hyakusoku H. Miyashita T. (2003). Postoperative electron-beam irradiation therapy for keloids and hypertrophic scars: Retrospective study of 147 cases followed for more than 18 months. Plast. Reconstr. Surg. 111 (2), 547–553. discussion 554-5. 10.1097/01.PRS.0000040466.55214.35 12560675

[B24] Park T. H. Rah D. K. Chang C. H. Kim S. Y. (2016). Establishment of patient-derived keloid xenograft model. J. Craniofac. Surg. 27 (7), 1670–1673. 10.1097/SCS.0000000000002901 27438448

[B25] Reinke J. M. Sorg H. (2012). Wound repair and regeneration. Eur. Surg. Res. 49 (1), 35–43. 10.1159/000339613 22797712

[B26] Renz P. Hasan S. Gresswell S. Hajjar R. T. Trombetta M. Fontanesi J. (2018). Dose effect in adjuvant radiation therapy for the treatment of resected keloids. Int. J. Radiat. Oncol. Biol. Phys. 102 (1), 149–154. 10.1016/j.ijrobp.2018.05.027 29970316 PMC7418482

[B27] Shen J. Lian X. Sun Y. Wang X. Hu K. Hou X. (2015). Hypofractionated electron-beam radiation therapy for keloids: Retrospective study of 568 cases with 834 lesions. J. Radiat. Res. 56 (5), 811–817. 10.1093/jrr/rrv031 26224888 PMC4577000

[B28] Shetlar M. R. Shetlar C. L. Hendricks L. Kischer C. W. (1985). The use of athymic nude mice for the study of human keloids. Proc. Soc. Exp. Biol. Med. 179 (4), 549–552. 10.3181/00379727-179-rc3 4022961

[B29] Shultz L. D. Ishikawa F. Greiner D. L. (2007). Humanized mice in translational biomedical research. Nat. Rev. Immunol. 7 (2), 118–130. 10.1038/nri2017 17259968

[B30] Song C. Wu H. G. Chang H. Kim I. H. Ha S. W. (2014). Adjuvant single-fraction radiotherapy is safe and effective for intractable keloids. J. Radiat. Res. 55 (5), 912–916. 10.1093/jrr/rru025 24801475 PMC4202283

[B31] Spaeth E. L. Dembinski J. L. Sasser A. K. Watson K. Klopp A. Hall B. (2009). Mesenchymal stem cell transition to tumor-associated fibroblasts contributes to fibrovascular network expansion and tumor progression. PloS one 4 (4), e4992. 10.1371/journal.pone.0004992 19352430 PMC2661372

[B32] Sruthi K. Chelakkot P. G. Madhavan R. Nair R. R. Dinesh M. (2018). Single-fraction radiation: A promising adjuvant therapy to prevent keloid recurrence. J. Cancer Res. Ther. 14 (6), 1251–1255. 10.4103/jcrt.JCRT_20_17 30488839

[B33] Wang X. M. Yu D. M. McCaughan G. W. Gorrell M. D. (2005). Fibroblast activation protein increases apoptosis, cell adhesion, and migration by the LX-2 human stellate cell line. Hepatology 42 (4), 935–945. 10.1002/hep.20853 16175601

[B34] Wang Y. Gan G. Wang B. Wu J. Cao Y. Zhu D. (2017). Cancer-associated fibroblasts promote irradiated cancer cell recovery through autophagy. EBioMedicine 17, 45–56. 10.1016/j.ebiom.2017.02.019 28258923 PMC5360585

[B35] Wang Y. Ma J. Zhang Z. Shen H. (2020). Combined surgical excision and electron external beam radiation improves the treatment of keloids: A descriptive study. Dermatol. Ther. 33 (4), e13494. 10.1111/dth.13494 32363669

[B36] Yan L. Cao R. Wang L. Liu Y. Pan B. Yin Y. (2015). Epithelial-mesenchymal transition in keloid tissues and TGF-β1-induced hair follicle outer root sheath keratinocytes. Wound Repair Regen. 23 (4), 601–610. 10.1111/wrr.12320 26036684

[B37] Yang X. Lin Y. Shi Y. Li B. Liu W. Yin W. (2016). FAP promotes immunosuppression by cancer-associated fibroblasts in the tumor microenvironment via STAT3-CCL2 signaling. Cancer Res. 76 (14), 4124–4135. 10.1158/0008-5472.CAN-15-2973 27216177

[B38] Yang Y. G. Sykes M. (2007). Xenotransplantation: Current status and a perspective on the future. Nat. Rev. Immunol. 7 (7), 519–531. 10.1038/nri2099 17571072

